# Challenges in Multimodal Pain Management During PCI in a Patient with Chronic Opioid Use: A Case Report

**DOI:** 10.51894/001c.164396

**Published:** 2026-08-01

**Authors:** Rashid Mossallam, Obaida Falah, Ahmed Chaudhary, Khalil Kamal

**Affiliations:** 1 Detroit Medical Center - Sinai Grace Hospital

## 45

### Introduction

Patients with opioid use disorder present unique challenges in procedural pain management due to opioid tolerance, which may render standard sedation and analgesia ineffective. Although multimodal analgesia strategies are commonly employed, they may still fail to provide adequate pain control during complex and high-risk procedures as percutaneous coronary intervention (PCI). Inadequate sedation can result in agitation or patient movement, increasing the risk of procedural complications. These challenges highlight the importance of careful pre-procedural planning and individualized sedation strategies, including consideration of deeper sedation or early airway control in select high-risk patients. This case illustrates the failure of multimodal analgesia in an opioid-tolerant patient undergoing PCI and raises the clinical question of whether preemptive intubation and deeper sedation should be considered to prevent life-threatening complications.

### Case Summary

We report the case of a 73-year-old woman with a history of coronary artery disease, diabetes mellitus, and opioid use disorder on chronic methadone therapy who presented with a non–ST-elevation myocardial infarction. She underwent elective coronary angiography via right femoral access due to radial artery disease. During the procedure, despite receiving multiple doses of opioids and sedative medications, the patient experienced uncontrolled pain and agitation. She subsequently moved abruptly and dislodged the femoral arterial sheath, resulting active hemorrhage. The patient required emergent endotracheal intubation and was taken urgently to surgery, where she underwent repair of two femoral arteriotomies and ruptured femoral pseudoaneurysm.

### Discussion

This case highlights the risks associated with unrecognized or underestimated opioid tolerance during procedural sedation. Standard moderate sedation may be insufficient in patients receiving chronic opioid therapy, increasing the risk of agitation and movement during invasive procedures. These events result in serious complications, particularly during vascular interventions requiring stable access. Early recognition of opioid tolerance and a multidisciplinary approach involving cardiology, anesthesia, and perioperative teams may allow for tailored sedation. In select cases, deeper sedation or preemptive airway control may reduce the risk of procedural complications.

### Conclusion

With chronic opioid use, standard procedural sedation may be inadequate. Careful pre-procedural risk stratification and individualized sedation planning including consideration of deeper sedation or prophylactic intubation—may improve procedural safety and reduce complication risk.

